# Morphology of conducting polymer blends at the interface of conducting and insulating phases: insight from PEDOT:PSS atomistic simulations†

**DOI:** 10.1039/d2tc03158b

**Published:** 2022-10-03

**Authors:** Hesam Makki, Alessandro Troisi

**Affiliations:** Department of Chemistry, University of Liverpool Liverpool L69 3BX UK h.makki@liverpool.ac.uk a.troisi@liverpool.ac.uk

## Abstract

Having phase-separated conductive and less-conductive domains is a common morphology in semiconducting polymer blends as it exists in the case of PEDOT:PSS, which is a representative example with a wide range of applications. In this paper, we constructed atomistic models for the interface between the PEDOT-rich (conductive) grains and the PSS-rich (less-conductive) phase through molecular dynamics simulations. Our models are obtained from experimentally relevant compositions, based on precise force field parameters, and through a robust relaxation procedure. We show that both PEDOT-rich and PSS-rich phases consist of PEDOT lamellae embedded in PSS chains. The size of these lamellae depends on the PEDOT concentration in each phase and our model predictions are in quantitative agreement with the experimental data. Furthermore, our models suggest that neither the phases nor the interfaces are entirely connected by π–π stacking. Thus, inter-lamellae tunnelling is essential for both intra- and inter-grain charge transport. We also show that a small increase (≈8 wt%) in the PEDOT concentration results in rather larger lamellae sizes, considerably more oriented lamellae, and slightly better inter-lamellae connectivity, which result in enhanced intra-grain conductivity. Moreover, we show how enhancing phase separation between PEDOT-rich and PSS-rich domains (similar to the effect of polar co-solvents), *i.e.*, pulling out PEDOT from the PSS-rich phase and adding it in the PEDOT-rich phase, highly enhances the intra-grain connectivity but decreases the inter-grain conduction paths through the interface. Our results explain how the marginal extra degree of phase separation (based on experimentally obtained values) could result in a great enhancement in the overall film conductivity.

## Abstract 2

Reliable atomistic models for the interface between conductive and less-conductive domains of PEDOT:PSS are provided through MD simulations and verified against the experimentally-obtained morphology.

## Graphical abstract

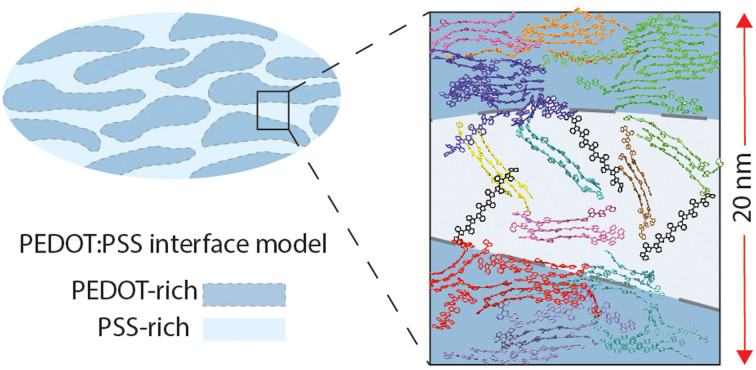

## 1. Introduction

Mixing conducting and insulating polymers results in organic conducting materials with enhanced processability and mechanical properties; therefore, a large fraction of conducting polymer materials consists of a nanophase-separated morphology,^1^ in which the conducting-polymer-rich phase carries out the charge transfer function and the softer insulating-polymer-rich phase facilitates materials processing and improves the desired mechanical properties.^2,3^ Poly(ethylene dioxythiophene):poly(styrene sulfonate) (PEDOT:PSS) is one of the most practically relevant conducting polymer blends, which is commercialized for a wide range of applications, *e.g.*, stretchable electronics,^4^ transparent conductive electrodes for organic solar cells and sensors,^5^ and organic electrochemical transistors.^6^ Besides, employing hydrophilic polymers (*i.e.*, PSS) in the insulating phase provides a pathway for ionic conduction while the electronic conduction takes place along the conducting-polymer-rich grains (*i.e.*, PEDOT-rich phase).^7^ This has particular applications in bioelectronics.^6,8^ Charge transfer inside the conducting-polymer-rich grains occurs quickly and efficiently and the bottleneck seems to be the inter-grain transfer,^9,10^ where the charge needs to transfer through a disordered region, *i.e.*, the PSS-rich phase, to reach another aggregated conductive grain.^11^

The phase organization of PEDOT:PSS has been extensively studied.^12–14^ The commercial material is a dilute dispersion of PEDOT : PSS (1 : 2.5 weight ratio) in water. After film application and water/co-solvent evaporation, a phase-separated morphology forms, where the PEDOT-rich (conducting) phase with a diameter of about 50–80 nm^12,14^ and the PSS-rich (less-conductive) phase with a size ranging from 3 to 10 nm,^15–17^ exist. It is worth emphasizing that other film preparation and treatment methods might lead to different morphologies so that the existence of similar size PEDOT-rich and PSS-rich phases has also been reported.^18^ An in-depth atomic force microscopy (AFM) analysis suggested that about 75 v% of the film consists of conductive grains and around 25 v% is occupied by the PSS-rich phase.^14^ X-ray scattering analyses quantified the concentration of PEDOT in conductive grains and the less-conductive phase^12^ based on the previous AFM results^14^ and showed that neither of the domains are pure. Rivany *et al.* determined that the PEDOT concentration ranges between 40 and 45 wt% in the PEDOT-rich domain and 37–29 wt% for the pristine and ethylene glycol modified samples.^12^ It is therefore to be noted that the weight fraction of PSS is greater than that of PEDOT in both phases and the “PEDOT-rich” term only denotes the larger concentration of PEDOT in the grains compared to the “PSS-rich” phase.

An important feature of PEDOT:PSS is that its conductivity varies tremendously, *i.e.*, from ∼2 to >4000 S cm^−1^, based on the film preparation and post-treatment methods.^19^ The significant enhancement in the conductivity is related to the morphological changes of the polymer blend as a result of the treatments.^20^ In general, as more PEDOT is pulled out from the PSS-rich phase (and added to PEDOT-rich grains) and the more PSS is washed out from the PSS-rich phase, the conductivity of the film increases. For instance, it is shown that using a co-solvent with a higher boiling point, *e.g.*, dimethyl sulfoxide (DMSO), leads to a larger and higher number of PEDOT-rich domains which provide smaller interchain distances and higher local ordering of lamellae.^21^ Also, using an ethylene glycol (EG) bath after DMSO treatment results in the partial removal of PSS from the film and decreases the inter-grain distances (suggested by the X-ray diffraction (XRD) experiment) and further increases the conductivity.^21^ Acid post-treatment is another way to remove PSS from the film and is shown to be highly effective in increasing the film conductivity as it creates a fibrillar structure to act as an interconnected conductive network;^22^ however, it has been shown that if it occurs on previously solvent-doped films, a considerable conductivity reduction will be observed.^10^ Combined IR and UV-vis spectroscopy, XRD, and conductive sensing AFM analyses revealed that although acid treatment on previously solvent-doped films increases the conductive domain sizes, it also increases the distance between the conductive domains (inter-grain distance); therefore, even if the intra-grain conductivity considerably increases, the overall film conductivity decreases due to the lower inter-grain connectivity.^10^ This shows that both the intra- and inter-grain morphologies play a role in the overall film conductivity.

A great deal of effort has been devoted to describe the conductive grains and PSS-rich phase morphologies and draw its connection with the film conductivity.^10,12,14,18,21,23^ For instance, it was shown that using co-solvent treatments, *e.g.*, DMSO or EG, results in a more significant phase separation between the conductive and non-conductive domains; therefore, π–π stacking and PEDOT chain ordering are enhanced and this results in enhanced conductivity.^14,21^ Later on, as mentioned earlier, Rivany *et al.* gave a quantitative estimation about the PEDOT concentration changes in both phases and showed that the change in concentration is really marginal (around 5 wt%);^12^ however, such a small change in concentration could significantly increase the overall film conductivity. Therefore, an important question has remained unsolved: how the very small PEDOT concentration changes in both phases result in two orders of magnitude increase in conductivity.^12^ Also, one expects that removing PEDOT from the PSS-rich phase and adding it to the conductive grains (*e.g.*, by co-solvent treatment) deteriorates the inter-grain connectivity (by the depletion of the PSS-rich phase of PEDOT chains) which is against the experimental observation that the overall film conductivity has been significantly enhanced. Thus, a model that can cover the intra- and inter-grain network connectivity so that it can explore the effect of PEDOT concentration is needed. Besides, the importance of the interfacial region between the two phases is not limited to the inter-grain charge conductivity. This interface is also the region in which ion transport takes place in bioelectronics materials, where a balance between ion and electron/hole transports is necessary.^7^ Thus, a reliable morphological model for this interfacial region could be tremendously informative for designing the ion/charge transport balance. Furthermore, despite the detailed above-mentioned models, there are still unclarities with respect to the PEDOT networks in both domains and their interface. For instance, it is unclear whether crystallites are entirely connected by a π–π stacking network and whether there is a large difference in the orientation of lamellae inside PEDOT-rich and PSS-rich phases.

Molecular dynamics (MD) simulations have been widely employed to provide realistic microscopic models of PEDOT:PSS and explain a variety of observations.^24–36^ The majority of these studies have focused on PEDOT:PSS in solution and elucidated the phase organization of PEDOT:PSS in water^33,35^ and the effect of co-solvents^27,30,31^ and ionic liquids^24,25,29,34^ on the phase separation between the conductive grains and the less conductive phase for PEDOT:PSS colloidal particles. Also, a few MD simulation studies modeled the PEDOT:Tos (tosylate) film morphology during^32,36^ and after drying^26^ (water evaporation) and computed charge mobility based on the MD models and showed good agreement between the model's predicted and experimentally obtained values.^26^ These studies prove that the MD simulation is a powerful tool to model the nano-phase organization of PEDOT:PSS; however, it has not been used neither to understand the effect of PEDOT concentration on the conductive network nor to model the interface between the PEDOT-rich and PSS-rich domains of PEDOT:PSS films, whose importance has been highlighted by the studies outlined above.

The goal of this work is to develop atomistic models for the PEDOT-rich and PSS-rich domains and their interface by MD simulations based on the experimentally obtained results so that the above-mentioned missing morphological details become clear. We particularly wish to understand the PEDOT network connectivity inside each phase and through the interface and address the effect of the degree of phase separation between the two domains on the intra- and inter-grain conductivity. Such a model should able to explain the significant enhancement of the charge conductivity as a result of a marginal phase-separation enhancement. The methodology, presented in the next section, focuses on demonstrating that the generated structures can be considered well equilibrated and reasonably representative. In the result, we will specifically analyze the lamellae size, lamellae orientation, the degree of π–π stacking, and the possible tunneling paths through the PEDOT-rich/PSS-rich interface.

## 2. Methods

**Fig. 1:**
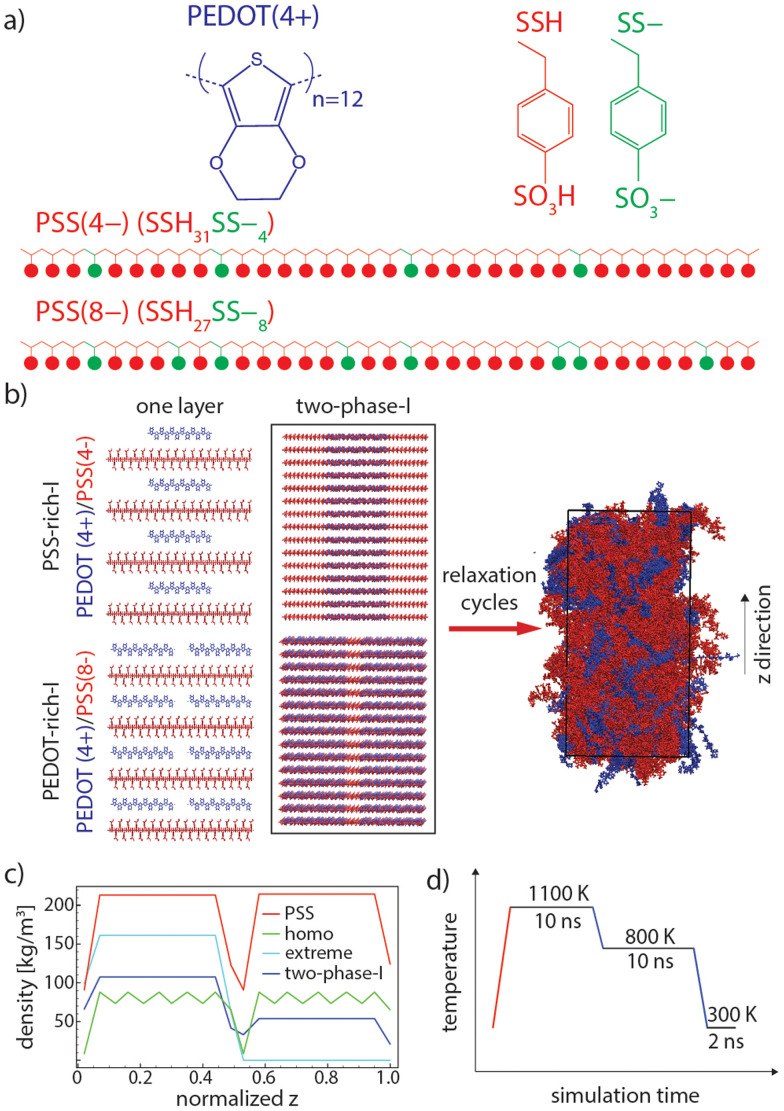
(a) Chemical structures of PEDOT and PSS. PSS has 35 repeat units in total, including 4 or 8 (depending on the PEDOT concentration) SS– and 31 or 27 SSH, respectively. An adequate number of SS– repeat units is placed randomly in each PSS chain. (b) Initial and relaxed configurations of “two-phase-I” (PSS and PEDOT are shown in red and blue, respectively). (c) PEDOT and PSS density profiles of “two-phase-I”, “homo”, and “extreme” initial configurations (see Table 1). (d) One cycle of relaxation (heating and cooling cycles are shown in red and blue, respectively).

Our method combines three components that are believed to exist in PEDOT:PSS:^35^ (i) PEDOT chains (here only in a bipolaronic state), (ii) styrene sulfonate (SS–, which represents deprotonated), and (iii) styrene sulfonic acid (SSH, which represents protonated) monomers of PSS (see Fig. 1(a)). Note that components (ii) and (iii) are subunits of all PSS chains and their molar ratio in each chain varies depending on the local concentration of PEDOT. Thus, the PEDOT-rich phase contains more SS– (which are neutralized by the positive charge of PEDOTs) compared to the PSS-rich phase.

### 2.1. Materials and force fields

PEDOT with 12 repeating units in a bipolaronic state (+4 charge) and PSS with 35 repeating units (−4 and −8 charges) were modeled based on the recently developed and accurately verified force field parameters generated for PEDOT^37,38^ and generalized amber force field (GAFF),^39^ respectively. Note that the −4 and −8 charges on PSS molecules were made by randomly replacing 4 and 8 SSH monomers with SS– ones, respectively (see Fig. 1(a)). Charge positions on PSS repeating units were randomly selected so that a statistically independent mixture of PSS chains with different repeating unit sequences was generated for each simulation box (see two examples of PSS chains in Fig. 1(a)). In this way, PSS chains with a similar total charge (*i.e.*, total number of SS– monomers) are not identical with respect to the sequence of deprotonated (SS–) and protonated (SSH) monomers in model chains, which is a reasonable representation of real PSS chains in PEDOT:PSS. It is also worth noting that the experimental molecular weight of PEDOT is estimated to be in the range of ≈1–2.5 kDa (7–18 repeating units) while PSS often has very large molecular weights (≈400 kDa).^13,40^ Note that the exact chain size of PEDOT cannot be determined since pure PEDOT cannot be synthesized without counterions.^41^ Thus, our model PEDOT size is reasonably representative while mimicking real PSS average size is not feasible for MD simulations. PSS chains with 35 repeating units with a contour length of around ≈11.3 nm are large enough to be the template for up to two PEDOT chains, with a contour length of ≈4.9 nm (which is the maximum PEDOT:PSS ratio in our models, see Table 1) and also provide enough randomness for inserting 4 and 8 negatively charged monomers in the chain. Besides, since the focus of this research is modeling the PEDOT network morphology, it is essential that PEDOT chains are in the range of the experimental molecular weight while keeping the PSS chains comparatively larger, as performed in other atomistic models.^11,37^

**Table 1:** Simulation labeling and details

Model category	Model names	PEDOT:PSS [wt%] (PEDOT : PSS [number of chains])	Box size [total number of atoms]
Large concentration difference (model type I)	(1) PEDOT-rich-I	52 (120 : 60)	60 600
	(2) PSS-rich-I	26 (60 : 60)	51 360
	(3) Two-phase-I	39 (120 : 60/60 : 60)	111 960
Small concentration difference (model type II)	(4) PEDOT-rich-II	43 (100 : 60)	57 520
	(5) PSS-rich-II	35 (80 : 60)	54 440
	(6) Two-phase-II	39 (100 : 60/80 : 60)	111 960
Hypothetical models	(7) HOMO (homogeneous PEDOT distribution)	39 (45 : 30/45 : 30)	55 980
	(8) Extreme (extreme PEDOT distribution)	39 (90 : 30/0 : 30)	55 980
	(9) Two-phase-I	39 (60 : 30/30 : 30)	55 980

It should also be noted that model PEDOT chains are monodispersed in this study, which might result in a slightly different distribution and the percolation behavior of the PEDOT network compared to the real conditions, as it was previously shown that polydispersity slightly increases the percolation threshold for conductive (fiber-like) particles^42^ and polymer chains^43^ by experimental and simulation studies. However, it is unfeasible to obtain an experimentally relevant polydispersity for PEDOT chains due to the lack of pure PEDOT polymerization, and accordingly, appropriate characterization of this material, as mentioned before.

### 2.2. Initial configurations

#### 2.2.1. Individual phases

Each phase was made by stacking 15 layers of the adequate number of bipolaronic state PEDOT chains inserted onto the PSS templates (with a specific charge for each phase, *i.e.*, −4 or −8, depending on the number ratio of PEDOT/PSS in each phase) so that the net charge of each layer was preserved at zero (see Fig. 1(b), “one layer”, where each layer of the PSS-rich phase is made of 4 PEDOT(+4) and 4 PSS(−4) and each layer of the PEDOT-rich phase is made of 8 PEDOT(+4) and 4 PSS(−8)). In this way, the initial configuration of simulation boxes remains as close as possible to the real condition, where EDOT oligomers polymerize alongside the PSS chains^12,28^ and the sulfonate anionic groups of PSS (SS– monomers, see Fig. 1(a)) are the counterions used to balance the doping charges.^44^ The remaining monomers of PSS will be in the form of SSH (see Fig. 1(a), red monomers), which is a reasonable occurrence upon the drying of PEDOT:PSS and the protonation of remaining SS– (*i.e.*, the ones that are not already neutralized by the neighboring positively charged PEDOT) by the transformation of hydronium groups to form SSH.

We constructed two model types, denoted as “I” and “II”; the first one with large concentration differences of 52 wt% and 26 wt% of PEDOT in the PEDOT-rich and PSS-rich phases, respectively, and the second one with smaller concentration differences of 43 wt% and 35 wt% of the PEDOT weight in the two phases. These models match the experimentally obtained PEDOT concentration ranges for PEDOT-rich and PSS-rich phases^12^ and could help us to understand the effect of the degree of phase separation between the phases on the conductive network.

#### 2.2.2. Two-phase models

The interface of PEDOT-rich and PSS-rich phases was made by overlaying the initial configuration of individual models (see Fig. 1(b), “two-phase-I” as an example). Accordingly, two interface initial configurations were made: (1) PEDOT-rich phase with 52 PEDOT:PSS wt% and PSS-rich phase with 26 PEDOT:PSS wt% (two-phase-I, see Table 1), and (2) PEDOT-rich phase with 43 PEDOT:PSS wt% and PSS-rich phase with 35 PEDOT:PSS wt% (“two-phase-II”, see Table 1). Note that the achievable simulation size does not allow the entire grains of the PDOT-rich phase to be simulated and this model will explore the interface with an area of ∼60 nm^2^ and a thickness of around 20 nm.

To ensure the validity of the relaxation procedure, we established a simulation protocol that produces similar equilibrium structures starting from different initial conditions. The different initial configurations have similar total PEDOT concentrations as the realistic model “two-phase-I” (see “homo” and “extreme” and “two-phase-I” information in Table 1), but with a different initial distribution of PEDOT chains along the *z* axis. First, a homogeneous distribution of PEDOT chains in the two (so-called) PEDOT-rich and PSS-rich phases was considered for the “homo” model (see Table 1), and second, an exaggerated interface, for which all the PEDOT chains are in the PEDOT-rich phase and there is no PEDOT in the PSS-rich phase of the box, was considered for the “extreme” model. Fig. 1(b) shows the PEDOT and PSS density profiles for the initial configuration of “two-phase-I” and the two control simulations (“homo” and “extreme”). As shown, the PSS chain distribution is similar for all three models. Note that the model sizes (total number of atoms) of control simulations were chosen to be smaller than the 6 main models (models 1–6 in Table 1) to reduce computational cost.

Simulation boxes were made with an initial density of 0.28 g cm^−3^ and packed to a density of around 1.40 g cm^−3^ with a similar algorithm as developed by Kong and Liu.^45^ It is worth noting that the bulk model dimensions after packing are 6–7 × 9–10 × 9–10 (in *x*[nm] × *y*[nm] × *z*[nm] format) and the two-phase model dimensions are 6–7 × 9–10 × 19–20 (in *x*[nm] × *y*[nm] × *z*[nm] format) depending on the PEDOT concentration. Thus, the thickness of the PSS-rich phase (9–10 nm in the *z* dimension) lies within the experimentally obtained range, *i.e.*, 3–10 nm.^15–17^

### 2.3. Relaxation procedure

PEDOT:PSS shows a high glass transition temperature (*T*_g_) in MD simulations (around 1050 K);^11^ therefore, equilibration of initial configurations at room temperature (around 300 K) is not feasible. Besides, there is a high chance of being kinetically trapped in unfavorable configurations if one performs equilibrations above *T*_g_ followed by a (direct) rapid cooling to 300 K, similar to many polymer glasses.^46^ Therefore, we used a “sub-*T*_g_ relaxation” protocol, as developed by Keene *et al*.,^11^ in which an intermediate annealing at 800 K (slightly below *T*_g_) was performed between the equilibration above *T*_g_ (at 1100 K) and at 300 K. A schematic of this procedure is depicted in Fig. 1(d). Several consecutive annealing steps were performed for each simulation and morphological parameters and the chain configuration has been monitored to ensure well-equilibrated models.

Relaxation and production simulations were performed under an *NPT* ensemble with a time step of 1 fs by using GROMACS. A 1.4 nm cutoff for Lennard-Jones and electrostatic interactions was used and all nonbonded interactions for 1–2 and 1–3 bonded pairs were excluded and a scaling factor of 0.5 was used for 1–4 bonded pairs. The V-rescale thermostat^47^ and C-rescale barostat^48^ were used for packing steps and the Nose–Hoover thermostat^49^ and Parrinello–Rahman barostat^50^ were employed for relaxation and production runs. The Verlet cut-off scheme was employed for non-bonded interactions and particle-mesh Ewald was used for long-range electrostatic interactions.

### 2.4. Analysis

#### 2.4.1. π–π stacked PEDOT lamellae

**Fig. 2:**
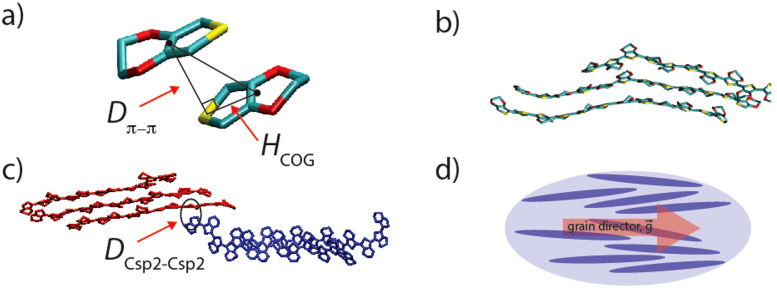
(a) A π–π stacked EDOT pair (carbon, oxygen and sulfur are shown in cyan, red, and yellow, respectively). *D*_π–π_ is the π–π stacking distance and *H*_COG_ shows the horizontal distance between the center of geometry, for two EDOTs in the π–π stacking interaction. (b) An example PEDOT lamella. (c) A non-π–π stacking contact point between two PEDOT lamellae (red and blue). *D*_Csp^2^–Csp^2^_ represents the distance between one sp^2^ carbon atom belonging to the red lamella and one sp^2^ carbon atom belonging to the blue lamella. (d) A schematic of the PEDOT chain orientation (blue ellipses represent PEDOT chains and red arrow shows the grain director).

To analyze the lamellae formed by PEDOT and the connectivity between domains, we identified π–π stacked EDOT pairs through the simulation trajectories. We defined the plane spanned by the heavy atoms of each EDOT monomer (through a least squares fitting) and recognized π–π stacked EDOTs on different chains that satisfy the following criteria: (1) the angle between two planes’ normal vectors is smaller than 10° (parallel EDOTs), (2) the π–π stacking distance (marked as *D*_π–π_ in Fig. 2(a)) is lower than 0.4 nm, and (3) the horizontal distance between the center of the geometry of each parallel pair (marked as *H*_COG_ in Fig. 2(a)) is lower than 0.5 nm. Last, all the PEDOT chains sharing at least one π–π stacked EDOT pair were considered to belong to a single PEDOT lamella (see Fig. 2(b) as an example).

#### 2.4.2. Inter-lamellar connectivity

To study the connectivity between lamellae, we compute the shortest distance between sp^2^ carbon atoms belonging to two lamellae (see Fig. 2(c), marked as *D*_Csp^2^–Csp^2^_). We considered two lamellae connected, to the effect of charge transport, if such a distance was shorter than a distance threshold (*d*_t_), which was varied in the typical range of electron tunneling (0.3–1.0 nm).

#### 2.4.3. PEDOT orientation parameter

The orientation of lamellae in individual phases and at the interface is an important morphological parameter with respect to charge transfer properties. We use a scalar parameter, which is typically used for uniaxial nematic liquid crystals to quantify the ordering of the molecules along a director:^51^1
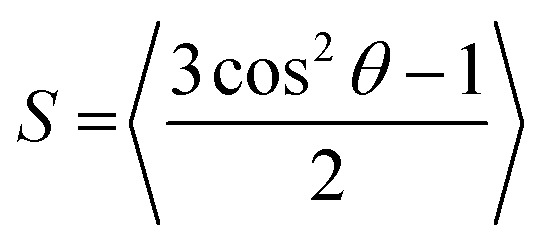
where *θ* is the angle between the PEDOT molecular axis and the grain director (see Fig. 1(g)) and the brackets represent the spatial and time average (over selected PEDOT chains during the simulation trajectory).

The codes that (i) generate the random PSS structure and topology files, (ii) calculate π–π stacked pairings, (iii) inter-lamellae connectivity, and (iv) PEDOT scalar orientation parameter (*S*) are all available in a public repository.^52^ Necessary input files for the codes and simulations are also provided.

## 3. Result

### 3.1. Equilibration verification

**Fig. 3:**
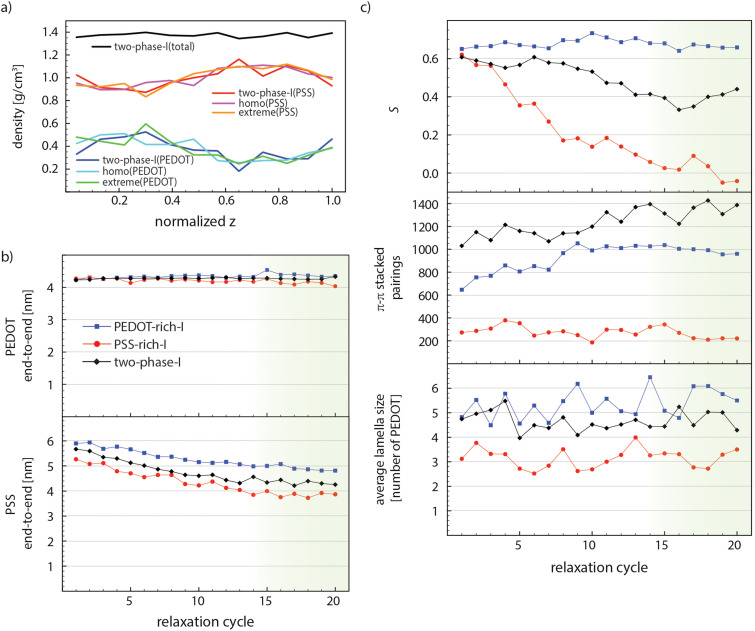
(a) PSS and PEDOT density profiles of “two-phase-I”, “homo”, and “extreme” models and the total density of “two-phase-I” after 10 relaxation cycles (compared with Fig. 1(b), the initial configurations). (b) PSS and PEDOT end-to-end lengths, (c) average lamella size, the number of π–π stacked pairings, and scalar orientation parameters (*S*) of “PEDOT-rich-I”, “PSS-rich-I”, and “two-phase-I”, through the relaxation process. The green shade shows the steady-state part.

A reliable morphological model created by the MD simulation needs a convincing equilibration procedure, particularly in the case of conducting macromolecules, which are associated with a wide range of relaxation time scales. The PEDOT:PSS film preparation in the experiment consists of multiple steps, *e.g.*, film formation, solvent evaporation at elevated temperatures, post treatments, and cooling down to room temperature, which take place in a rather long and unfeasible time scale for MD simulations. However, it is feasible to obtain a relaxed structure by defining reasonable initial configurations and performing necessary relaxation simulations until the structural parameters remain steady upon further annealing cycles (see one annealing cycle in Fig. 1(c)). The strong electrostatic interactions between negatively charged PSS and positively charged PEDOT chains force them to remain next to each other and the great tendency of PEDOT chains to form π–π stacked lamellae pushes them to form stacked domains surrounded by PSS chains. Our first check for the sufficiency of the relaxation process is to evaluate if relaxation simulations provide enough thermal energy (mobility) for PEDOT chains to easily move according to the above-mentioned interactions. Therefore, we performed two control simulations with markedly different initial PEDOT distributions through the *z* dimension compared to “two-phase-I” (read Section 2-2-2 and see Fig. 1(b)). Fig. 3(a) shows that after 10 relaxation cycles the PEDOT and PSS density profiles of all three simulations collapse into a single graph. This suggests that the relaxation scheme is fairly successful in providing enough mobility for PEDOT chains to freely move through the interface simulation box to find the equilibrium morphology.

As the second measure, we computed the conformational properties of polymer chains, *i.e.*, the end-to-end length and the radius of gyration (*R*_g_), for “PEDOT-rich-I”, “PSS-rich-I”, and “two-phase-I” models during the relaxation process. Fig. 3(b) illustrates the change in the average end-to-end lengths of PEDOT and PSS for these models. PEDOT tends to remain stretched during the whole process (it preserves 86% of its contour length after 20 relaxation cycles), which is expected due to the very rigid nature of conjugated polymers^53,54^ and rather short (12 repeat-unit) PEDOT chains. Also, a slightly larger PEDOT end-to-end length of “PEDOT-rich-I” (compared to “PSS-rich-I”) is due to the larger percentage of individual PEDOT chains (that do not stack in a lamella) for “PSS-rich-I” and they tend to slightly bend (apparently) because of their shorter persistence length. The PSS end-to-end distance shows a slight decay from cycle 1 to cycle 14 and then fluctuates around a constant value for all models. Note that the steady state value for “PEDOT-rich-I” is around 38% of its contour length. PSS chains are more stretched in the PEDOT-rich phase, which is due to the higher concentration of PEDOT that exerts larger stress on PSS templates and hinder their entropic tendency towards the coiled conformation. The “two-phase-I” value lies between the two phases’ magnitudes for both PSS and PEDOT end-to-end lengths. This suggests that polymers belong to each phase preserve their configuration even at the interface. Note that *R*_g_ follows a similar trend for all models and the results are depicted in Fig. S1 in the ESI.†

Morphological parameters are expected to change until equilibrium is reached and the relaxation time associated with each parameter might differ due to its nature. Fig. 3(c) shows the average size of PEDOT lamellae (the average number of PEDOT chains in each lamella), the total number of π–π stacked pairings (the average number of EDOT pairs in the model), and the PEDOT scalar orientation parameter *S* (see eqn (1)), respectively, of “PEDOT-rich-I”, “PSS-rich-I”, and “two-phase-I”, during relaxation. The average size of lamellae fluctuates around a constant value for each simulation, during the whole relaxation process. The lamella size is larger in the case of the PEDOT-rich phase, and in the case of “two-phase-I”, it falls between the two bulk simulations’ magnitudes. The considerable fluctuation of lamella size also indicates that PEDOT chains leave and join their lamellae during relaxation cycles. Also, the total number of EDOT pairs for “PEDOT-rich-I” and “two-phase-I” increases linearly and stabilizes (roughly) after cycle 14. The simultaneous increase in the total number of EDOT pairings and the constant average lamella size throughout the relaxation process suggest that PEDOT chains slide back and forth to find the maximum (equilibrium) amount of π–π stacking with other chains in the lamellae throughout the relaxation process. Last, the ordering of PEDOT lamellae (including individual PEDOT chains) in the grain direction, characterized by the scalar orientation parameter, is approximately constant through the whole relaxation time for “PEDOT-rich-I”, while this value exhibits a considerable and gradual decrease of the “PSS-rich-I” case. The initial configurations for both phases and the interface model consist of fully aligned chains towards the grain director (see Fig. 1(b)), which results in *S* = 1.5. This value decreases to around 0.65 for all cases right after the first relaxation cycle. While “PEDOT-rich-I” retains this value throughout the whole relaxation simulation, PEDOT chains in the “PSS-rich-I” model move towards a completely disordered morphology (*S* ≈ 0) which is reached after 14 relaxation cycles This, again, suggests that the PEDOT chain orientation is not hindered due to the lack of dynamics during relaxation. Similar relaxation was performed for “PEDOT-rich-II”, “PSS-rich-II”, and “two-phase-II” models and the results are presented in Fig. S2 in the ESI.† It is worth noting that we attempted to relax the starting configurations with differently oriented PEDOT and PSS chains but the convergence towards equilibrium was too slow so we did not pursue it.

**Fig. 4:**
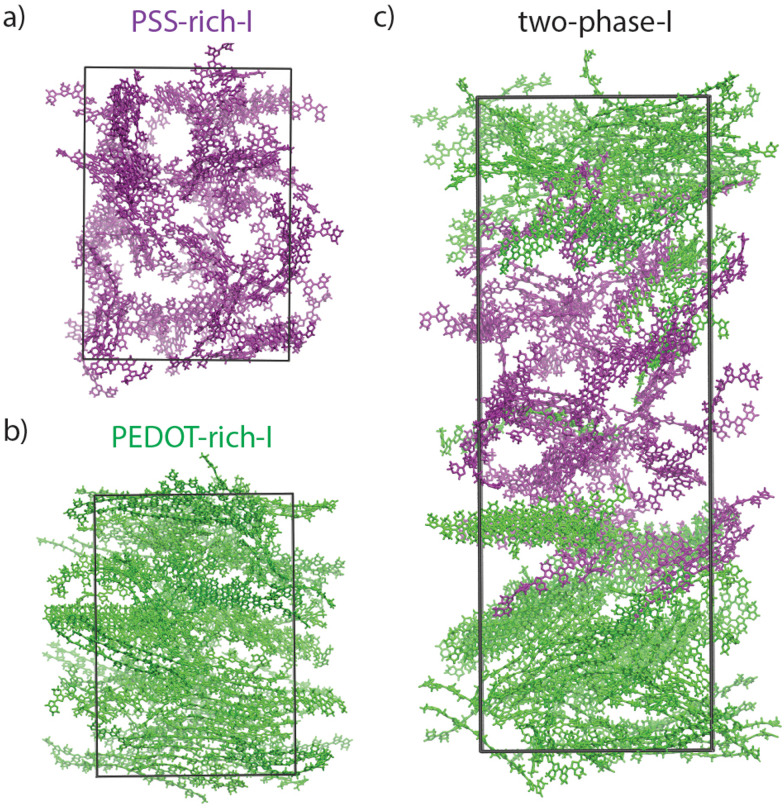
PEDOT snapshots (PSS chains are removed for better visualization) of “PSS-rich-I” (a), “PEDOT-rich-I” (b), and “two-phase-I” (c), at the end of 20 relaxation cycles. Note that PEDOT chains initially belonging to PSS-rich and PEDOT-rich phases are shown in purple and green, respectively.

Fig. 4(a)–(c) show the snapshots taken at the end of the last (20) relaxation cycles for “PSS-rich-I”, “PEDOT-rich-I”, and “two-phase-I”, respectively. PSS chains are removed for a better visualization (similar to all snapshots shown in the following figures) and all the PEDOT chains in the PEDOT-rich and PSS-rich phases are colored in green and purple, respectively. These snapshots (visually) indicate the isotropic PEDOT network in “PSS-rich-I” (Fig. 4(a)) and strongly oriented chains in “PEDOT-rich-I” (Fig. 4(b)) models. In the case of the “two-phase-I” model (Fig. 4(c)), one could see that PEDOT chains from both phases (purple and green colored) share lamellae at their interface, which suggests π–π stacked coupling between the two phases, but even after 20 relaxation cycles (a total of around 0.52 μs simulation time) a very limited number of PEDOT chains have interchanged between the two phases.

### 3.2. PEDOT network in individual phases and through the interface

**Fig. 5:**
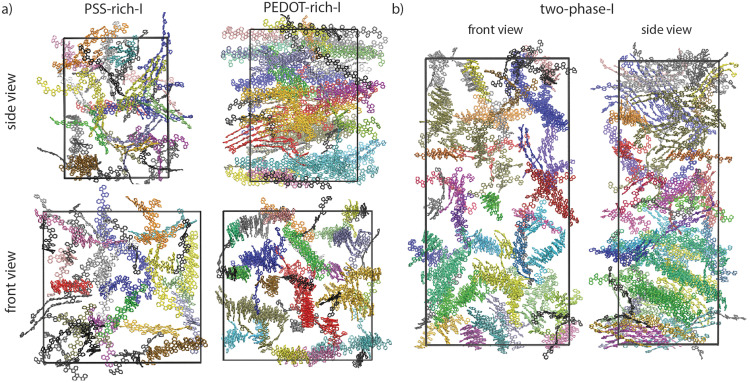
(a) Lamellar organizations of “PSS-rich-I”, “PEDOT-rich-I”, and (b) “two-phase-I” simulations after relaxation from front and side views. Each lamella is uniquely colored for each simulation and individual chains are colored in black for all cases.

The PEDOT lamellar organizations of bulk and interface simulations are illustrated in Fig. 5(a) and (b). Each color in each equilibrated simulation box represents an individual lamella, as characterized by π–π stacking analysis (see Section 2-4-1). This means that differently colored lamellae do not share any π–π stacked pair; therefore, neither in individual phases nor through the interface of the two phases is there entire connectivity by π–π stacking. Note that the average size of lamellae in each phase was previously quantified and is shown in Fig. 3(c). In fact, our models predict an average 5–6 PEDOT chain crystallite size in the PEDOT-rich phase (see Fig. 3(c)) which is in quantitative agreement with previously driven GIWAXS results,^18^ where the size of the nano-crystallite PEDOT domains inside the PEDOT-rich domain was estimated to be 1–2 nm and consist of 5–6 PEDOT chains embedded in the amorphous PSS matrix.

**Fig. 6:**
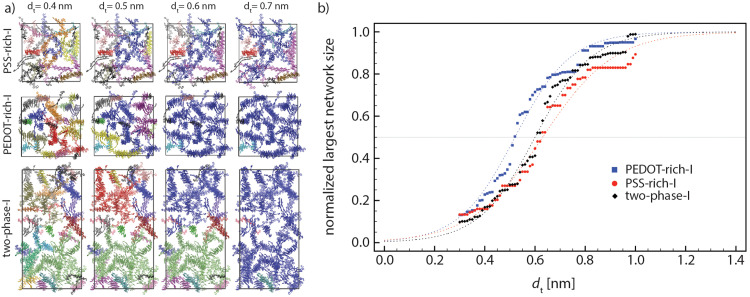
(a) Inter-lamellae connectivity as characterized by *D*_Csp^2^–Csp^2^_ (see Fig. 2(c)) < *d*_t_. The PEDOT chains represented in the same color at each (Csp^2^–Csp^2^) distance threshold (*d*_t_) represent an interconnected PEDOT network (as *d*_t_ increases, the size of the largest interconnected PEDOT network increases). (b) Normalized largest network size of PEDOT chains, as a function of *d*_t_ for “PEDOT-rich-I”, “PSS-rich-I”, and “two-phase-I” models. All values are averaged over the last 5 relaxation cycles. The dashed lines are fitted functions (eqn (2)) on the data.

In general, larger lamellae transport charge more efficiently. Also, according to our models, there is no continuous π–π stacking network across the grain and electron conduction should process through the connection between lamellas that cannot be characterized as π–π stacking. A strong electronic interaction between close (∼0.35 nm) sp^2^ carbons between non-π–π stacked molecules is ubiquitous in high mobility molecular crystals^55^ with the strength of the interaction and electron tunneling probability decreasing approximately one order of magnitude every additional 0.1 nm.^56^ The inter-lamellae connectivity is explored by estimating the number of possible charge transfer sites between all (previously) recognized lamellae. The sp^2^ carbon atoms belonging to each lamella can become close to sp^2^ carbon atoms of other lamellae without being in a π–π stacking interaction (see Fig. 2(c) as an example). Thus, the distances between all sp^2^ carbons belonging to each lamella with the adjacent ones *D*_Csp^2^–Csp^2^_ (as schematically shown in Fig. 2(c)) were calculated. The connectivities of all lamellae and individual chains were analyzed by setting a range of distance thresholds (*d*_t_) for *D*_sp^2^–sp^2^_, *i.e.*, *d*_t_ = 0.3–1.0 nm with 0.01 nm steps. The graphical representation and (normalized) quantitative measures were employed to picture the total inter-lamellae connectivity within the bulk of each phase and through the interface. Note that by increasing the *d*_t_, possible longer range tunneling between PEDOT chains have been considered. Thus, the size of the largest PEDOT network (through inter-lamellae connectivity) as a function of *d*_t_ could give an indication of the PEDOT network connectivity, which determines the overall film conductivity. This analysis illustrates the percolative behavior of PEDOT chains inside the film as a well-known behavior in conductive polymer composites^57^ so that the smaller the *d*_t_ at which the majority of PEDOT chains are (considered) connected, the better the overall film conductivity. Thus, in Fig. 6(a), we redefined the color maps so that all connected lamellae (and individual chains) by sp^2^ carbons closer than the specified *d*_t_ (*i.e.*, *d*_t_ = 0.4, 0.5, 0.6 and 0.7 nm) are colored similarly. Therefore, Fig. 6(a) illustrates the PEDOT lamellar connectivity of individual phases (*i.e.*, “PSS-rich-I” and PEDOT-rich-I”) and “two-phase-I” models at the final relaxation cycle in a way that for each *d*_t_, the unicolored PEDOT chains are considered in contact. Obviously, more PEDOT lamella are considered connected as the connecting threshold increases. The value of *d*_t_ at which the majority (>50%) of PEDOT chains become interconnected is a direct measure of the longest tunneling distance for the charge carrier and a simple morphological model correlated with the resistivity.^11^

Fig. 6(b) depicts the normalized largest PEDOT network size as a function of *d*_t_ for “PEDOT-rich-I”, “PSS-rich-I”, and “two-phase-I” models during the 5 last relaxation cycles (each point shows the average over the last 5 relaxation cycles). As shown, this quantitative percolation analysis also suggests a higher degree of connectivity for PEDOT-rich grains and the corresponding magnitudes for the interface lie between the two phases’ values. The data points were fitted by a sigmoid function:2
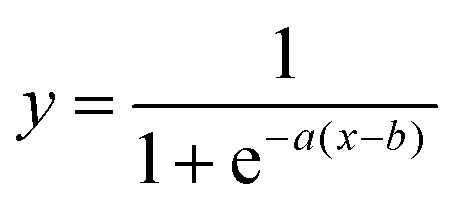
where *a* and *b* are constants. The mid-point of the fitted function (*b*) introduces the distance threshold at which 50% of PEDOT chains are (considered) connected. We calculated these values for “PEDOT-rich-I”, “two-phase-I”, and “PSS-rich-I” models as 0.52, 0.60, and 0.63 nm, respectively. Therefore, our models propose a larger lamella size and a better inter-lamellae connectivity for the conducting grains, while these values for the interface lie between the values associated with individual phases.

Percolation analysis that has previously been performed for PEDOT:Tos (tosylate) models as a function of the PEDOT chain length (by a two-component models consisting of positively charged PEDOT and negatively charged tosylate molecules at different water contents, *i.e.*, 0–43 wt%)^26^ showed that the size of π–π stacked connected PEDOT clusters depends on the PEDOT chain length so that by increasing the chain length, the morphology evolves from well-defined but disconnected PEDOT crystallites to a less ordered but efficient network of π–π stacked PEDOTs throughout the material.^26^ However, our models suggest that entirely π–π stacked connected PEDOT chains do not exist in the case of PEDOT:PSS (see Fig. 5), in the range of PEDOT concentrations of this study. It should be noted that (i) the concentration of PEDOT in the PEDOT:Tos study was markedly different and (ii) one expects that the polymeric nature of PSS plays a significant role in the PEDOT:PSS morphology. Thus, the rather different percolation behavior could be due to the different morphologies resulting from the two models.

### 3.3. Effect of the PEDOT concentration

As mentioned earlier, Rivany *et al.* estimated the PEDOT concentration change in conductive and less-conductive phases and showed that with a marginal change (about 5–8%) in PEDOT concentration between the two domains, two orders of magnitude increase in conductivity is achieved.^12^ Thus, we built up models with different PEDOT concentrations for PEDOT-rich, PSS-rich, and accordingly, their interface models (models type II, see Table 1), based on the relevant experimental data, to elucidate the effect of PEDOT concentration on network morphology and possible conductive pathways through the interface. We study the effect of the PEDOT concentration in two stages. First, we consider how the enrichment of PEDOT modifies its connectivity in terms of π–π stacking, inter-lamellae coupling, average lamella size and orientation. Second, we look specifically at the effect of a reduced PEDOT concentration at the interface between two PEDOT-rich grains.

**Fig. 7:**
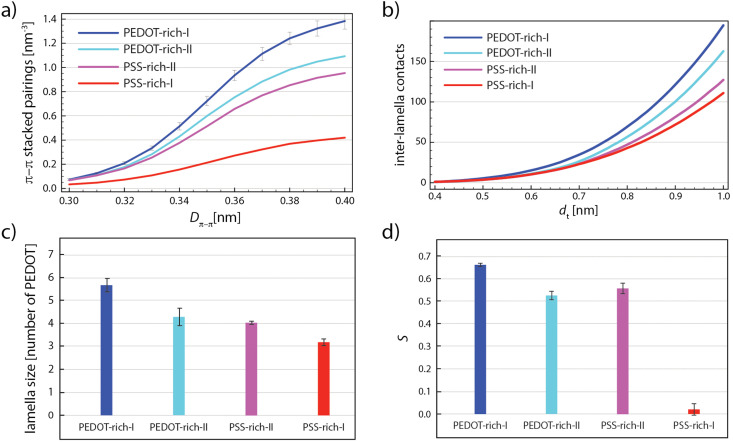
(a) The cumulative number of π–π stacked EDOT pairs per nm^3^ as a function of *D*_π–π_. (b) The number of inter-lamella contacts normalized by the total number of lamellae as a function of *d*_t_. (c) The average lamella size and (d) scalar orientation parameter (*S*) of all individual phase models. The error bars show the standard deviation of the results for the five last relaxation cycles.

The PEDOT network parameters are plotted in Fig. 7(a)–(d), for different individual phase models in a decreasing order of PEDOT concentrations: “PEDOT-rich-I” (52 wt% PEDOT:PSS), “PEDOT-rich-II” (43 wt% PEDOT:PSS), “PSS-rich-II” (35 wt% PEDOT:PSS), and “PSS-rich-I” (26 wt% PEDOT:PSS). Note that all the values in Fig. 7(a)–(d) are averaged over the last 5 relaxation cycles. Fig. 7(a) shows the number of EDOT–EDOT π–π stacked pairings per unit volume [nm^−3^] as a function of the π–π stacking distance (*D*_π–π_ in Fig. 2(a)) for each individual phase model. Note that Fig. 7(a) shows that the characteristic π–π distance can be identified with the inflection point, in the narrow range of 0.35–0.36 nm, in agreement with the experiment^58^ and other simulation studies^25^ (see the non-cumulative distribution curves in Fig. S3 in the ESI†). As expected, the number of π–π stacked pairings increases with the PEDOT concentration, which is one of the reasons for a better conductivity of highly concentrated grains as obtained by solvent/acid (post)treatments.^58^

Fig. 7(b) illustrates the number of inter-lamellae contacts (including individual chains) for *d*_t_ = 0.4–1.0 nm. Note that the total number of contacts are divided by 4 (each EDOT has 4 sp^2^ carbon atoms) and by the number of lamella (including individual chains). One observes that the average number of contacts between PEDOT lamellae is not significantly different for different models with varying PEDOT concentrations, apart from a considerably higher number for the “PEDOT-rich-I” model.

Fig. 7(c) depicts the effect of PEDOT concentration on the average lamella size. An expected observation is that the average lamellae size decreases with the decreasing PEDOT concentration. Of course, larger lamellae expect to be more efficient in charge transport and enriching conductive grains with PEDOT will result in a better conductivity. Fig. 7(d) depicts the lamellae orientation as a function of the PEDOT concentration. As shown, *S* only slightly decreases by the decreasing PEDOT concentration from 52 wt% (PEDOT-rich-I) to 35 wt% (PSS-rich-II) but by further decreasing the PEDOT concentration to 26 wt% (PSS-rich-I), a significant transition from a substantially orientated (“PSS-rich-II”) to a completely anisotropic morphology (“PSS-rich-I”) occurs.

**Fig. 8:**
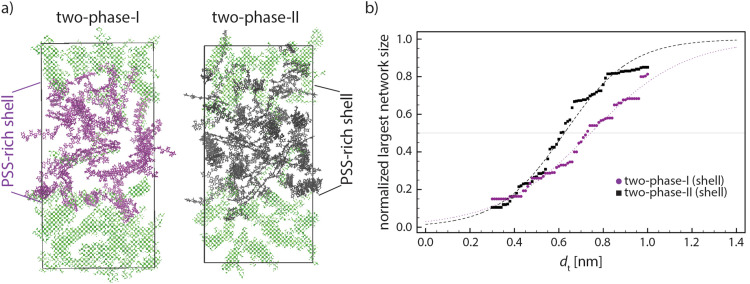
(a) Snapshots of “two-phase-I” and “two-phase-II” in which purple and black PEDOTs show the PSS-rich phase of “two-phase-I” (higher phase separation between two phases) and “two-phase-II” (lower phase separation between two phases), respectively. The PEDOT-rich phase of both models is shown in (transparent) green. (b) Average normalized largest network for “two-phase-I” and “two-phase-II” models. The dashed lines are the fits by eqn (2). The largest network size data are the average values of the last 5 relaxation cycles for each model.

As the data in Fig. 7 suggest, a better intra-grain conductivity is expected by increasing the degree of phase separation between PEDOT-rich and PSS-rich phases due to the larger lamella size, higher degree of crystal orientation, and less inter-lamella distances because of the increase in PEDOT concentration inside the conductive grains. Fig. 8(a) shows the final snapshots of “two-phase-I” (largest difference of the PEDOT concentration in the two phases) and “two-phase-II” (smaller difference of the PEDOT concentration). Thus, the inter-lamellae connectivity in PSS-rich phases represents the degree of inter-grain connectivity for the two interfaces. Fig. 8(b) depicts the change in the size of the largest PEDOT network inside the PSS-rich phase of the two interface models as a function of the distance threshold *d*_t_. The reduced concentration of PEDOT in the interface of the “two-phase-I” model increases by ∼0.1 nm the distance threshold *d*_t_ where >50% PEDOT are connected, or, in other words, impose a longer charge tunneling distance to connect the conductive PEDOT-rich grains. This indicates that, in parallel with the enhancement of the PEDOT lamellar size, orientation, and connectivity within the conductive grains due to the phase-separation, the inter-grain connectivity inside the PSS-rich phase is deteriorated. It is worth noting that the tunneling rate in the condensed phase of organic materials decreases with distance following an exponential relationship ≈exp(−*βd*_t_) with the tunneling attenuation factor *β* typically in the range of 0.3–0.9 [Å^−1^].^56^ Furthermore, the value of *β* was recently estimated to be close to the lower boundary for PEDOT in the PSS matrix.^11^ Thus, an increase of the tunneling distance of around 0.1 nm to achieve percolative transport, caused by the reduced PEDOT concentration in the PSS-rich phase, would result in the relevant tunneling rate to decrease only by a factor of 0.74 (≈exp(−0.3 × 1)). Considering the much larger size of the PEDOT-rich grains (50–80 nm)^13,16^ compared to the PSS-rich thickness (3–10 nm),^13,16,17^ a small increase of the resistivity in the PSS-rich phase has a minimal effect on the overall resistivity. Accordingly, our model suggests that the enhancement of ordering and lamellae connectivity within the conductive grains when their PEDOT concentration is increased (see Fig. 7) is likely responsible for the experimentally observed enhancement in the overall film conductivity:^12,21^ the small increase in tunneling distances within the interface is more than compensated by the improved conductivity within the conductive grains.

**Fig. 9:**
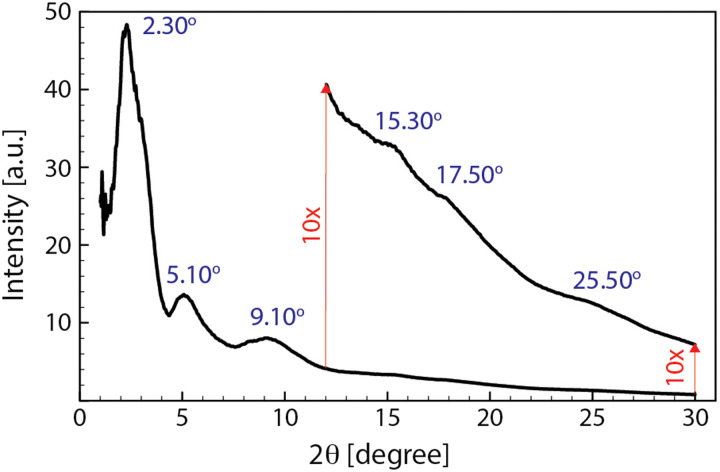
The simulated diffractogram of the two-phase-I model calculated by Debyer code60 for the final snapshot of the relaxation trajectory. The intensity of the pattern above 12 degrees is enlarged (10×) to magnify the peaks for better visualization.

As it is clear in the snapshots of our models (see Fig. 8(a)), the lamellae orientations are not considerably correlated as seen in other simplified models^12,23,59^ so that our models suggest that one cannot simplify the conductive gains as iso-oriented lamellae embedded by amorphous regions. This might explain the rather broad peaks appearing in the X-ray diffraction pattern of PEDOT:PSS.^21^ To compare the morphology of our model with the experimental analysis, we simulated the XRD diffractogram of the final snapshot of the two-phase-I model by using the Debyer code.^60^Fig. 9 shows that the simulated diffractogram predicts six dominant peaks, which is fully in line with the experimental PEDOT:PSS XRD pattern.^10,21^Table 2 shows the range of 2*θ* values of the six peaks obtained experimentally and from our models. The similarity between the simulation and experimental values reflects the accuracy of our models in predicting the PEDOT:PSS morphology.

**Table 2:** Experimental and simulated (two-phase-I) XRD major peaks

Experimental XRD peaks (2*θ*)^10,21^	3.8–4.1	6.7–8.1	10–11.2	13.8	17.2	25.50–26
Simulated XRD peaks (2*θ*)	2.30	5.10	9.10	15.30	17.50	25.50

Furthermore, one of the most important properties of PEDOT:PSS films is that they strongly swell in water.^61^ In the ESI† (Section S4), we have shown that our model is capable of reproducing the effect of water penetration although this phenomenon requires the fine tuning of additional aspects of the simulations (*e.g.*, variable protonation state during water diffusion).

## 4. Conclusions

The interfaces of the conducting-rich and non-conducting-rich domains of PEDOT:PSS were modeled by atomistic MD simulations. Our models have been designed to reproduce experimentally relevant compositions, based on reliable force field parameters, and through a robust equilibration scheme (at least 0.52 μs of annealing simulations). In agreement with crystallographic experiments, we found that both domains and their interfaces are made by a few nanometer size PEDOT lamellae embedded in the PSS matrix so that the size of PEDOT crystallites depends on the local PEDOT concentration (ranging from 3 to 6 PEDOT per lamella). The interface models also suggest that the PEDOT chains belonging to either (conductive or less-conductive) domain share some lamellae at their interface, which suggest π–π stacked coupling between the two phases. Also, we found out that the nanometric lamellae are not connected by an entire π–π stacked network in neither of the domains. One possible connecting path could be the close vicinity between the sp^2^ carbon atoms of PEDOT chains belonging to different lamellae.

The bulk and interface models predict a larger lamellar size, considerably more oriented lamellae, and a better interconnected PEDOT network for conductive grains, compared to the less-conductive phase. In this way, these models explain why a considerable improvement in the intra-grain conductivity occurs by a marginal enhancement in phase separation between PEDOT-rich and PSS-rich domains. Also, our models show that such a phase-separation decreases the interfacial connectivity of the two adjacent conductive grains due to the dilution of the PSS-rich phase of PEDOT chains, but the intra-grain morphological enhancement has a much larger effect so that a significant increase in overall film conductivity due to the enhancement of phase separation is expected. All in all, our models show to be capable of predicting morphological changes due to altering processing parameters and providing new insights into the morphology–conductivity relationship. These models and our methodology can be used to understand many atomistic-resolution properties at the interface between the conductive and non-conductive phases of semiconducting polymers such as the effect of additives (*e.g.*, solvents and ionic liquids) on the molecular morphology of the interface, ion transport for bioelectronics at the interface region, and interfacial morphological changes due to mechanical deformation for stretchable electronics.

Last, it should be noted that due to the wide diversity of PEDOT:PSS preparation and treatment methods, various morphologies exist for this material. Thus, the aim of our modeling is not to provide a universal picture of the PEDOT:PSS morphology based on the exact concentrations reported by Rivany *et al.*,^12^ but is to establish a methodology to enable the understanding of the morphology as a function of important parameters such as PEDOT concentration at the interface of PEDOT-rich and PSS-rich phases.

## Conflicts of interest

There are no conflicts to declare.

## Supplementary Material

TC-010-D2TC03158B-s001
