# Supplementary material for: Morphology of conducting polymer blends at the interface of conducting and insulating phases: insight from PEDOT:PSS atomistic simulations
Source: J Mater Chem C Mater. 2022 Oct 3;10(42):16126–37. doi: 10.1039/d2tc03158b (PMC9632246; doi:10.1039/d2tc03158b)
Supplement: TC-010-D2TC03158B-s001 [file TC-010-D2TC03158B-s001.pdf]

Electronic Supplementary Material (ESI) for Journal of Materials Chemistry C.
This journal is © The Royal Society of Chemistry 2022

Supplementary Information for:

Morphology of Conducting Polymer Blends at the Interface of Conducting and Insulating Phases: Insight from PEDOT:PSS Atomistic Simulations

Hesam Makki[§*], Alessandro Troisi[§]

[§]Department of Chemistry, University of Liverpool, Liverpool L69 3BX, U.K.

*Email: h.makki@liverpool.ac.uk, a.troisi@liverpool.ac.uk

S1- Radius of gyration (R_g) of PEDOT and PSS chains during relaxation

R_g of PSS and PEDOT chains as a function of relaxation cycles (in a similar presentation to Figure 3b (end-to-end length vs relaxation cycle) in the manuscript) is plotted in Figure S1. The green shaded area shows the steady-state part.

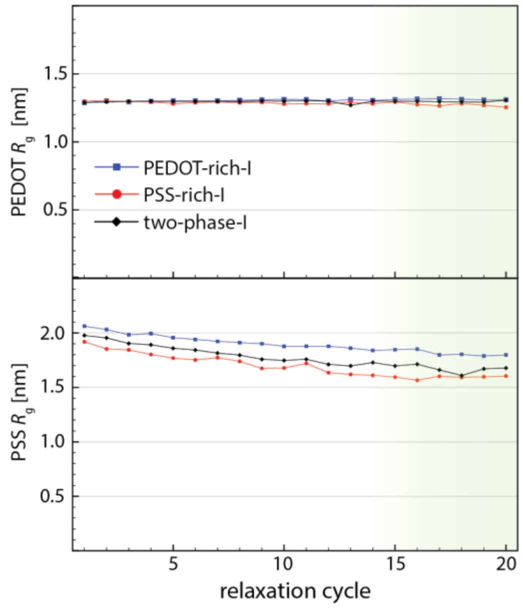

Figure S1. PSS and PEDOT R_g as a function of number of relaxation cycles for “PEDOT-rich-I”, “PSS-rich-I” and “two-phase-I” models.

S2- Relaxation process for “PEDOT-rich-II”, “PSS-rich-II”, and “two-stage-II”

The end-to-end length of PEDOT and PSS (Figure S2a) and other morphological parameters (Figure S2b), i.e., lamellae size, number of π-π stacked pairings, and the scalar orientation parameter *S* (as defined in Equation 1 in the manuscript), as a function of relaxation cycles are shown in Figure S2. Again, the green shaded area shows the steady-state part.

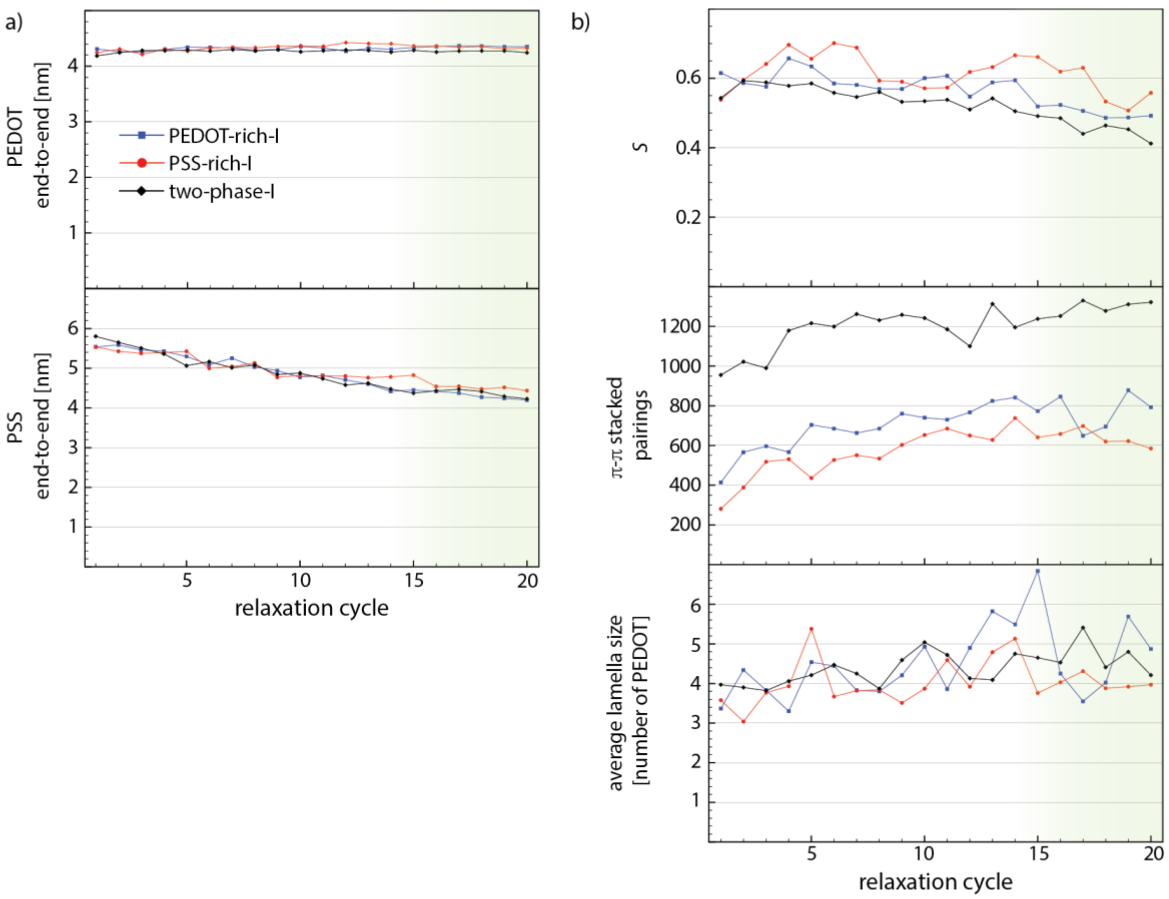

Figure S2 a) PSS and PEDOT end-to-end lengths, b) average lamella size, number of π-π stacked pairings, and scalar orientation parameters (S), for “PEDOT-rich-II”, “PSS-rich-II”, and, “two-phase-II”, through the relaxation process. The green shade shows the steady-state part.

S3- Non-cumulative distribution of π-π stacking distance

The distribution of π-π stacking distance $D_{\pi\text{-}\pi}$ (as described in Figure 2a in the manuscript) for all six main models are shown in Figure S3. The maximum lies between 0.35 and 0.36 nm.

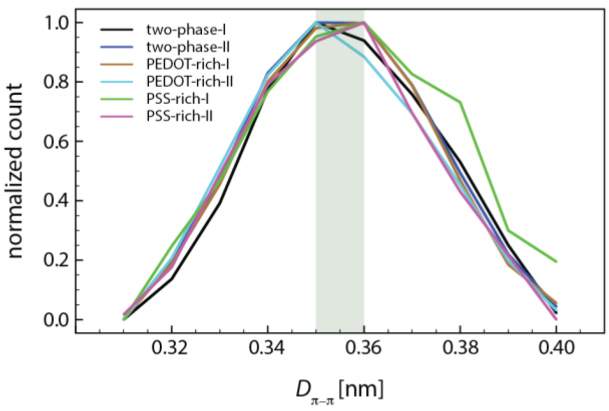

Figure S3. Normalized (between 0 and 1) distribution of π-π stacking distance $D_{\pi\text{-}\pi}$ for all six main models.

S4- Water intake behaviour of two-phase-I model

We generated an initial structure of water/polymer/water by placing 4nm thick (previously relaxed SPC/e) water layers on the top and bottom of a relaxed two-phase-I model (see Figures S4, the snapshots in panel a). After energy minimization and initial relaxations, we performed two simulations: (i) 500 ns at 300K and 1 bar and (ii) 50 ns at 1100 K (above T_g) and under NVT followed by a 100 ns simulation at 300 K and 1 bar under NPT. The snapshots (Figures S4, panel b for 300 K and panel c for 1100 K/300 K) and density profiles of water (Figure S5) in z direction during the simulations for both cases are shown. Note that we removed polymer chains in the snapshots of Figure1 b and c for a better visualization of water diffusion. As is clearly evident, the water swelling and intake is taking place at both temperatures; however, due to the glassy nature of this polymer at 300 K, the water diffusion is rather slower as compared to 1100 K (one might need a several-μs simulation to see the steady-state of the wet film at 300K). Note that the 100 ns simulation at 300 K after water diffusion at 1100 K does not result in any phase separation between polymer and water, which emphasizes on the miscibility of our PEDOT:PSS models and water at 300 K.

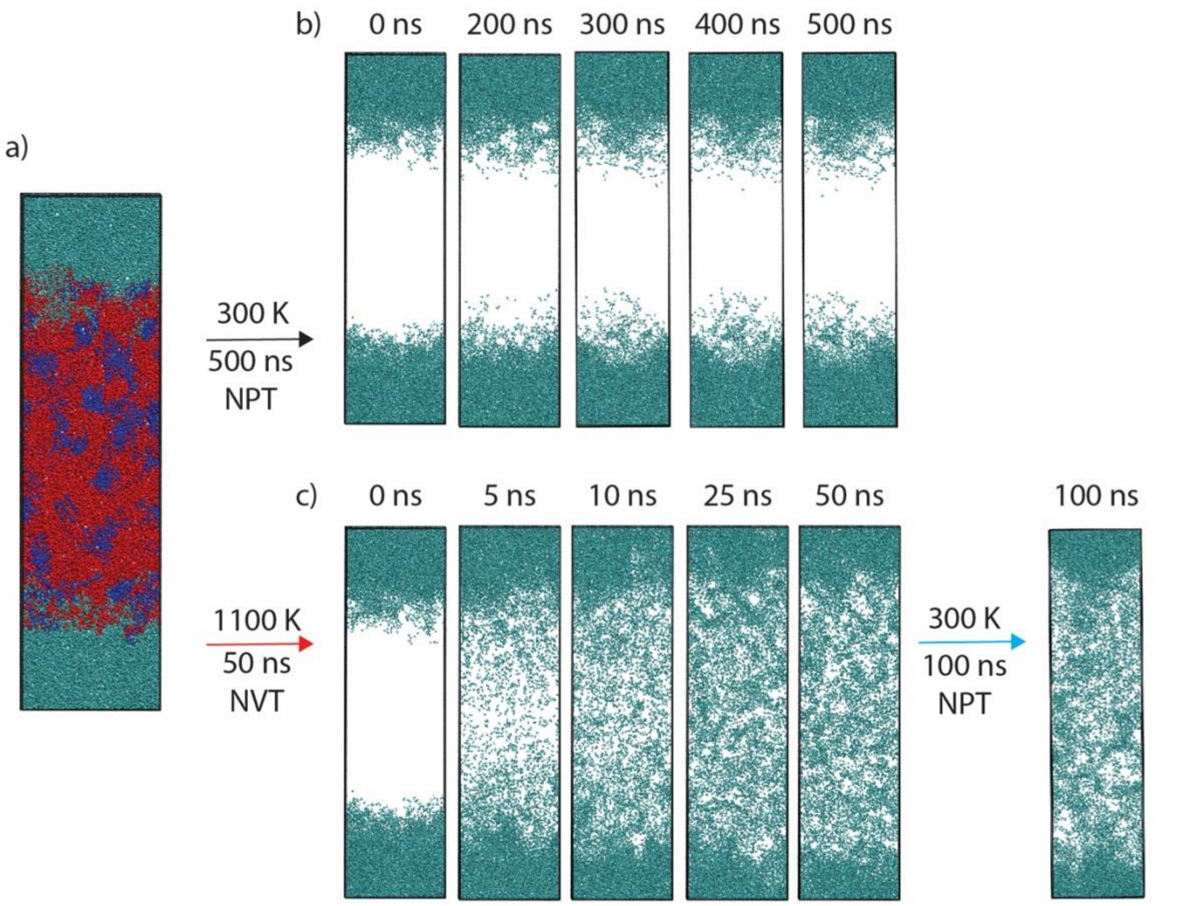

Figure S4. Water diffusion into two-phase-I model. a) Initial structure (after energy minimization and a short equilibration). b) Water diffusion at 300 K during a 500 ns simulation. c) Water diffusion at 1100 K during a 50 ns simulation followed by a 100 ns simulation at 300 K. Note that polymer chains are removed for snapshots shown in b and c.

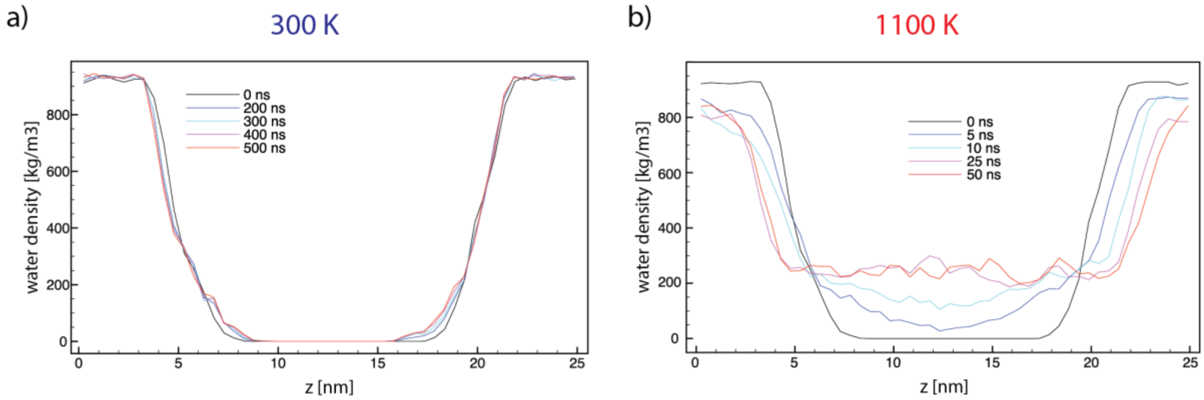

Figure S5. Density profiles of water for a) 300 K simulation and b) 1100 K simulations.
